# The role of global health partnerships in vaccine equity: A scoping review

**DOI:** 10.1371/journal.pgph.0002834

**Published:** 2024-02-22

**Authors:** Charnele Nunes, Martin McKee, Natasha Howard

**Affiliations:** 1 Faculty of Public Health and Policy, London School of Hygiene and Tropical Medicine, London, United Kingdom; 2 Saw Swee Hock School of Public Health, National University of Singapore and National University Health System, Singapore, Singapore; Institute of Development Studies, UNITED KINGDOM

## Abstract

The emergence of global health partnerships (GHPs) towards the end of the twentieth century reflected concerns about slow progress in access to essential medicines, including vaccines. These partnerships bring together governments, private philanthropic foundations, NGOs, and international agencies. Those in the vaccine field seek to incentivise the development and manufacture of new vaccines, raise funds to pay for them and develop and support systems to deliver them to those in need. These activities became more critical during the COVID-19 pandemic, with the COVAX Facility Initiative promoting global vaccine equity. This review identifies lessons from previous experiences with GHPs. Findings contribute to understanding the emergence of GHPs, the mechanisms they leverage to support global access to vaccines, and the inherent challenges associated with their implementation. Using Arksey and O’Malley’s method, we conducted a scoping review to identify and synthesise relevant articles. We analysed data thematically to identify barriers and opportunities for success. We included 68 eligible articles of 3,215 screened. Most (65 [95%]) were discussion or review articles describing partnerships or programmes they supported, and three (5%) were commentaries. Emerging themes included policy responses (e.g., immunisation mandates), different forms of partnerships arising in vaccine innovation (e.g., product development partnerships, public-private partnerships for access), and influence on global governance decision-making processes (e.g., the rising influence of foundations, diminishing authority of WHO, lack of accountability and transparency, creation of disease silos). If global health partnerships are to maximise their contributions, they should: (1) increase transparency, especially regarding their impacts; (2) address the need for health systems strengthening; and (3) address disincentives for cooperative vaccine research and development partnerships and encourage expansion of manufacturing capacity in low and middle-income countries.

## Background

The COVID-19 pandemic highlighted the scale and nature of global health inequity. Although safe and effective vaccines were developed in record time, the world’s richest countries retained many of the limited initial supplies, leaving billions in the Global South unprotected. In response, a coalition of governments and others created the COVAX Facility Initiative, co-led by the Coalition for Epidemic Preparedness Innovations (CEPI), Gavi, the Vaccine Alliance (Gavi), and the World Health Organization (WHO), working with the United Nations Children’s Fund (UNICEF) as major delivery partner. Gavi administers the facility [1]. COVAX is the mainstay of efforts to achieve global vaccine equity [2–4]. Still. it has limitations, with concerns about its ability to achieve its goal to “ensure that people in all corners of the world will get access to COVID-19 vaccines once they are available, regardless of their wealth” [5]. It builds on the experiences of its partners, in particular Gavi’s procurement model and CEPI’s work on vaccine development and manufacturing [6]. It is based on the principle of global solidarity, including self-financing and funded countries. The former group, which comprises high (HICs) and upper-middle-income (UMICs) countries, commit to supporting vaccine development and manufacturing and procuring sufficient vaccines to cover 20% of the world’s population.

COVAX is an example of a “global health partnership” (GHP), defined in 2004 by the United Kingdom (UK)’s Department for International Development (now FCDO) as “collaborative relationship[s] among multiple organisations in which risks and benefits are shared in the pursuit of a shared goal” [7, 8]. GHPs are seen as a vehicle for “collaborative transnational research and action for promoting health for all” [9] and include “advocacy partnerships”, “research and development (R&D) partnerships”, “access partnerships”, and financing partnerships”. Examples include the International AIDS Vaccine Initiative (IAVI), the Malaria Vaccine Initiative (MVI), the Global Alliance for Improved Nutrition and the Global Fund to Fight AIDS, Tuberculosis and Malaria (GFATM), and Gavi.

The rise of these partnerships was primarily driven by philanthropic foundations such as the Rockefeller Foundation and the Bill and Melinda Gates Foundation (BGMF) around the turn of the millennium. Gavi, created in response to concerns that, by the end of the 20^th^ century, immunisation rates were beginning to plateau in many parts of the world [10], is among the best-known of these partnerships. It seeks to increase vaccine access in low (LICs) and some middle (MICs) income countries. 80% of Gavi’s funding comes from governments, especially Canada, USA, UK, and the European Union (EU), and 20% from the sector, especially the BMGF [11]. Gavi used advance market commitments (AMCs), essentially contracts guaranteeing an agreed volume and prices of future purchases, to incentivise the development of new vaccines needed in LICs. Other mechanisms include product development partnerships (PDPs), which seek to accelerate research and development of new products for use in LMICs. The Rockefeller Foundation employed this approach in the mid-1990s to support development of an HIV vaccine [12, 13], leading to the creation of IAVI, in 1996.

The COVID-19 pandemic will not be the last. Although some lessons have already emerged, they will take time to digest. Therefore, to facilitate this learning process, we aimed to identify what lessons COVAX could learn from previous experiences with vaccine GHPs. Our objectives were to: (i) describe the emergence of GHPs in global health and identify why these partnerships were used as a model to achieve goals; (ii) identify the different forms GHPs in this field take and examine their strengths, limitations, and drawbacks, paying particular attention to the balance of costs and benefits to the parties involved; and (iii) summarise fundamental critiques of GHPs’ influence on global health decision-making processes.

## Methods

We conducted a scoping review using Arksey and O’Malley’s method [14], as refined by Levac et al., [15] which includes: identifying the research question; identifying relevant articles; selecting articles; charting/extracting data; synthesising; and reporting findings. We employed a broad research question and search strategy to include as many relevant articles as possible [16].

### Identifying the research question

Our research question, to also inform a broader study of GHPs, was: “What lessons can we learn from previous experiences with vaccine global health partnerships?”

### Identifying relevant articles

First, we searched six databases systematically (i.e., MEDLINE, EMBASE, Global Health, EconLit via Ovid, Web of Science, Scopus) in November 2021, using keywords and related MeSH terms and subject headings pertaining to global health partnerships and vaccines: (global public private partnership* or global health partnership* or global health initiative* or public private cooperation* or public private partnership* or public private sector cooperation* or public private sector partnership* or public private alliance* or private finance initiative* or product development partnership* or project finance*) AND (vaccin* or immuni* or immunization programs/ or vaccination coverage/).

We simultaneously conducted an iteratively refined Google Scholar search based on terms related to ‘public-private partnerships’ and ‘vaccines.’ We updated the database and Google Scholar searches in May 2023 to capture recent publications. Lastly, we backward-searched references of eligible articles [17].

### Article selection

After removing duplicates, CN screened titles and abstracts, then remaining full texts in EndNote 20 to assess compliance with eligibility criteria. Eligible article topics and outcomes were description of either GHPs’ vaccine innovation strategies strengths and weaknesses and any financial mechanisms (e.g., AMCs), or GHPs’ achievements or failures when promoting vaccine production or delivery. Given the focus on global vaccine coverage, country-specific case studies were excluded unless they discussed or compared more than one region or country. Article types included research articles and commentaries that included primary or secondary research data (e.g., policy analyses, systematic or scoping reviews, discussion papers). We had no geographical or language restrictions.

### Extracting data

We extracted relevant data under the following headings: lead author, publication year, location, intervention type and comparator (if any), duration, participants, study aim, methodology, and findings.

### Synthesising and reporting

As Arksey and O’Malley do not specify how to synthesise findings [15, 18], after summarising characteristics of eligible literature, we adopted a deductive thematic approach as described by Braun and Clarke [19] to elicit implications for policy, practice, and further research.

## Results

### Literature scope

**Fig 1:**
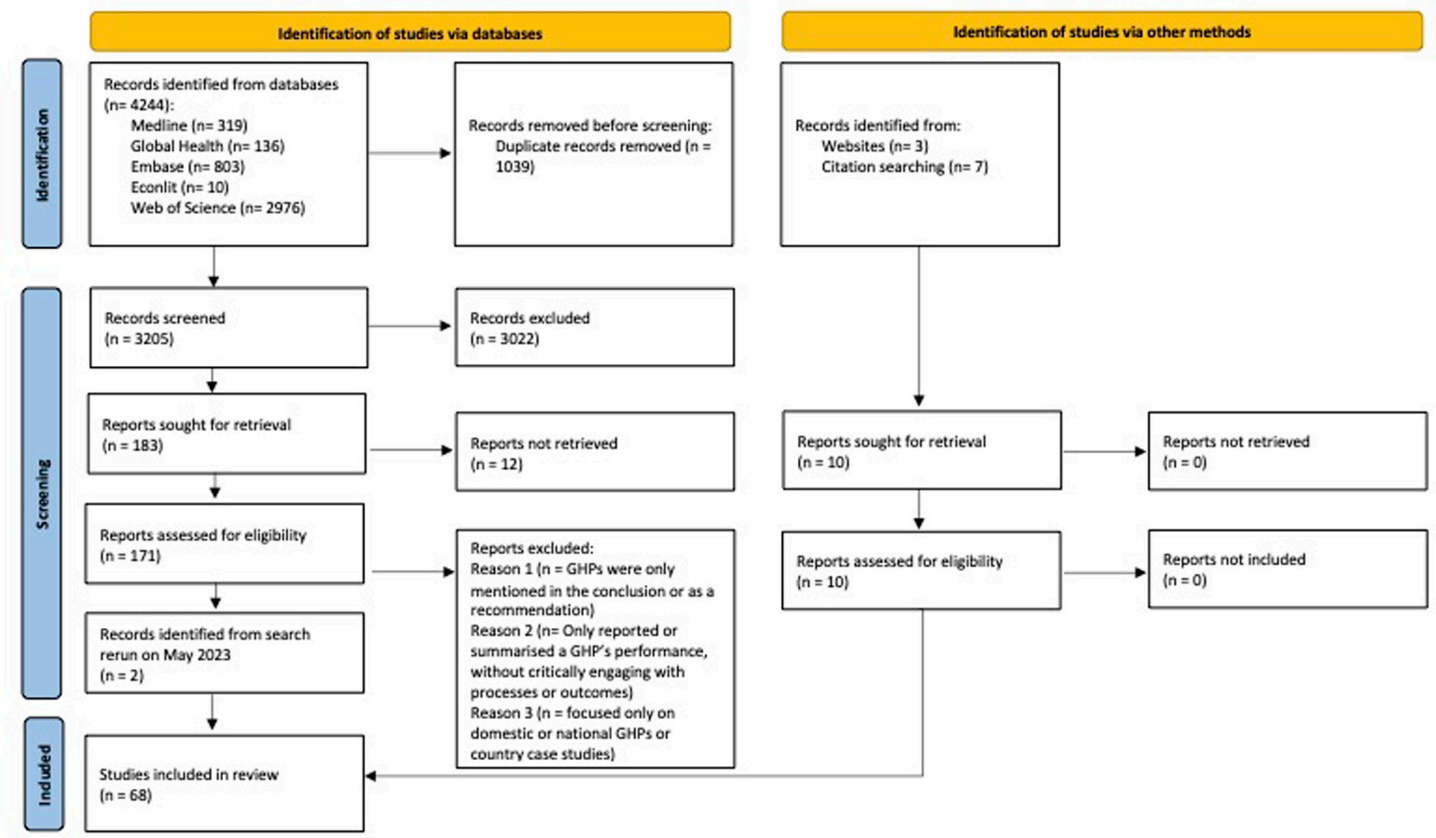
PRISMA flow diagram for scoping reviews.

Fig 1 shows we included 68 eligible articles of 3,215 initially identified (66 in the initial search and 2 added when we reran the search in May 2023) [20]. Of these, 58 were from databases and 10 from Google Scholar and citations. All were published in English. Most (65 [95%]) were review or discussion articles describing GHPs or GHP-supported initiatives, and three (5%) were commentaries.

### Thematic findings

Findings are organised under three deductive themes: (i) emergence of global immunisation partnerships in supporting global goals and interventions; (ii) different partnership types for vaccine innovation and access; and (iii) critiques of GHP influences on decision-making processes in global governance.

#### Emergence of global immunisation partnerships in supporting global goals and interventions

**Fig 2:**
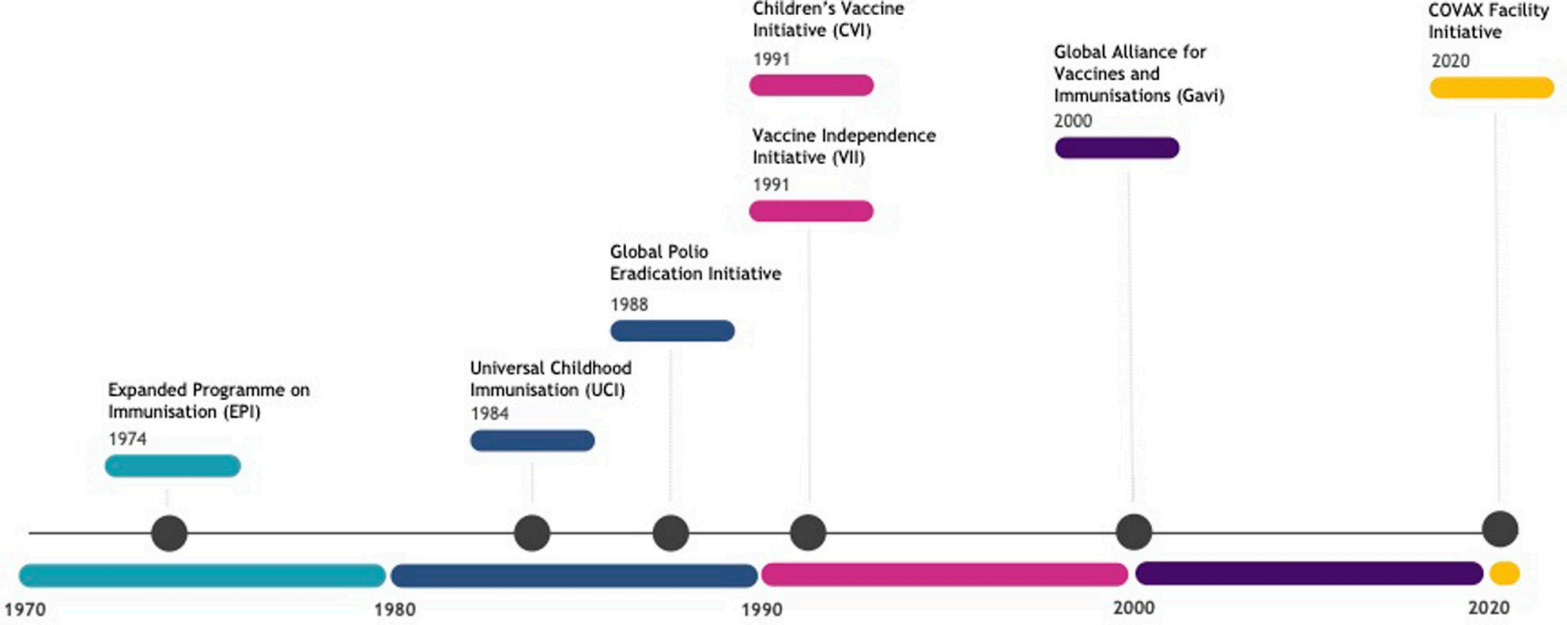
Timeline of global immunisation mandates.

*Evolution of global immunisation mandates and growing donor fatigue*. Five articles examined the evolution of global immunisation policies [21–25], starting with the global Expanded Programme on Immunisation (EPI), launched by WHO in 1974 to accelerate immunisation against six diseases at a time when less than 5% of the world’s children were immunised. EPI built on the success of smallpox eradication and, by 1990, had administered Bacille-Calmette-Guerin (BCG), polio, and measles vaccines to 80% of children under 13 months old globally [21, 22]. Hardon and Blume [21] examined five subsequent vaccine initiatives: (1) Universal Childhood Immunisation (UCI), launched in 1984 by WHO and UNICEF to accelerate EPI; (2) the Global Polio Eradication Initiative launched in 1988; (3) the Vaccine Independence Initiative (VII), launched in 1991 by UNICEF to overcome temporary budget shortfalls and maintain supplies; (4) the Children’s Vaccine Initiative (CVI), also launched in 1991, to promote new vaccine technologies and determine the feasibility of a single-dose multivalent vaccine; and (5) Gavi (formerly the Global Alliance for Vaccines and Immunisations) launched in 2000. Fig 2 provides a timeline of global immunisation mandates and launches of these initiatives, ending with the launch of COVAX in 2020.

Several authors identified donor fatigue in the 1990s as a factor in UNICEF’s creation of VII [21, 23]. Its importance was that, unlike its predecessors, it encouraged LMIC governments to take on greater responsibility for meeting vaccine needs, supporting them through subsidies for purchases. These governments were required to establish domestic vaccine budgets as a condition of participating in the pooled procurement mechanism, although UNICEF absorbed the risk of payment defaults. VII succeeded in increasing MICs’ self-reliance but less so for LICs.

*The rise of GHPs as brokers between public and private actors*. Four articles linked the origins of GHPs to events in the late 1980s and early 1990s [10, 21, 25, 26], when a series of developments coincided, including détente following the collapse of the USSR and the resulting reduction of decades of rivalry between superpowers competing for influence in poorer countries, the emergence of HIV, and the growth of public-private partnerships in sectors such as health and education in high-income countries (HICs).

GHPs were seen as a means of concentrating efforts at scale and raising large sums of money that could be spent on increasingly innovative vaccine technologies [10, 21, 25, 26]. CVI was co-sponsored by WHO, UNICEF, the United Nations Development Programme (UNDP), the World Bank, and the Rockefeller Foundation, and had substantially greater private sector involvement than its predecessors. This approach was initially very appealing to donors. However, tensions soon arose, particularly as European donors felt it was overly focused on technical solutions reflecting commercial interests [21]. CVI did develop improved vaccine technologies using PDPs but failed to mobilise needed resources to deliver and sustain immunisation programmes [21, 26]. These developments occurred alongside growing emphasis on health as a driver of economic growth, exemplified by the 1993 World Development Report [27], and the importance of ensuring interventions were cost-effective. The Millennium Development Goals provided further stimulus, leading governments to find new ways of funding development that gave private actors increasing influence in global health policy decision-making [10, 22]. Increasingly, public-private partnership structures came to be seen as essential in any global response.

Four articles described how this new worldview led to GHPs acting as either integrators or brokers of knowledge, working toward a common goal to achieve vaccine mandates, often focusing on technical progress [28–31]. This brokering role was particularly prominent in intellectual property issues, with GHPs acting in “*mediating asymmetric economic relationships between HICs and LMICs*” while ensuring that “*these initiatives do not seek to explicitly challenge the rules of international trade*, *global finance or the [IP] rights regime that underpin the neoliberal system of economic governance*, *viewed by many as the basis of continuing North-South intra-country inequities*” [31]. Thus, proposed solutions worked within the “rules of the game” and did not challenge established power relationships.

Gavi was launched in 2000. As a successor to CVI, it embodied and reinforced the priority to expand and sustain uptake of cost-effective vaccines and develop new vaccine technologies. Countries became eligible for Gavi support if their Gross National Income (GNI) per capita was ≤US$ 1,000 over the previous three years [21]. Unlike its predecessors, Gavi was not led by a UN agency but governed by a board of 16 institutional members. Five are permanent (i.e., BMGF, the Vaccine Fund, WHO, UNICEF, World Bank), and 11 rotate to reflect different constituencies (i.e., LMICs [2 seats], HICs [3], NGOs [1], LMIC industry [1], HIC industry [1], foundations [1], technical health institutes [1], research and academia [1]). Jean Stéphenne, former president of SmithKline Biologicals, which produced the combined DTP-hepatitis B vaccine, outlined the conditions for industry participation at the first Gavi Partners’ meeting in Noordwijk, the Netherlands, in 2000. These included a guarantee of “‘*reasonable prices’*, *support for a credible and sustainable market*, *respect for international property rights*, *a tiered pricing system including safeguards against re-exporting products from lower-income countries to high-priced markets*, *and a prohibition on compulsory licensing*” [21]. Gavi made aid contingent on increased immunisation coverage performance, which overlooked weaknesses in health information systems in most applicant countries. This was already recognised in the UCI initiative, with Hardon and Blume [21] reporting problems in Ghana, Mozambique, and Tanzania, none of which could provide the required data on DTP3.

### Different partnership types for vaccine innovation and access

#### GHPs for product development

Six articles described PDP successes in encouraging innovation [26, 32–36]. PDPs used public money to reduce risks for private companies undertaking high-cost research while subsidising development and commercialisation processes [26, 37]. Examples include the Drugs for Neglected Diseases Initiative (DNDi), the International AIDS Vaccine Initiative (IAVI), and the Malaria Vaccine Initiative (MVI). However, Sunyoto argued that their successes cannot remove the need to transform the current research and development system to address unmet health needs [36].

The most significant criticism of the PDP model is that it seeks to reconcile two conflicting principles, that the public sector should be responsible for addressing population needs within a finite budget and that the private sector should maximise profits [32, 34, 35]. It also avoids challenging an intellectual property regime that reinforces the private sector’s considerable power over pricing [32, 36]. This means other financial mechanisms are needed to procure products for LMIC markets [24, 38]. Some authors also noted how PDPs confer additional benefits to manufacturers, allowing them to enhance public reputation as they portray themselves as acting in the public interest to solve complex problems [26, 36]. A further concern about PDPs is their overall lack of local stakeholder engagement. Some have attempted to engage local stakeholders in decision-making processes related to regulatory processes and clinical trial management [22, 39]. However, there is still some way to go to incorporate the co-production approaches increasingly used in other settings [40].

PDPs support the growth of globally competitive vaccine manufacturers in LMICs [31, 37, 39, 41]. The expansion of manufacturing capacity in LMICs facilitates regional development of vaccine technologies, prompting LMICs to partner in the vaccine innovation process [39]. This occurs through public and private-sector investments, with PATH and BMGF playing essential roles as supporters and facilitators [37]. For example, the Serum Institute of India (SII) is now the largest producer of vaccines globally by volume. BMGF subsidised much of SII’s portfolio, including rotavirus, human papillomavirus (HPV) and pneumococcal vaccines [31]. Moran and Stevenson found that “*the Foundation’s leadership believes the world needs several companies like the SII with global distribution capacity to meet demand projections and diversify risks*” [31].

Five articles contended that PDPs fulfil their intended purpose by developing new drugs, vaccines, and other technologies despite longstanding state and market failures [28–30, 39, 42]. For example, by the end of 2004, global health partnerships, including PDPs, were responsible for nearly 75% of neglected disease drug development and negotiated conditions for IP rights [28, 31, 32]. DNDi developed eight new treatments for malaria, leishmaniasis, Chagas Disease, sleeping sickness, paediatric HIV, and hepatitis C. Hayter and Nisar [39] noted that PDPs developed strategies to motivate company and university participation in instances where IP protection was a barrier to collaborative governance. Licensing plans included co-assigning patents with commercial partners or not patenting at all. Chataway et al. [28] and Moran and Stevenson [31] highlighted IAVI’s attempt to monitor how the financial consequences of intellectual property rights should be shared between public and private actors by brokering a negotiated agreement in which private actors retain IP rights and IAVI retains the right to obtain licences to contract other vaccine manufacturers if the original company should decline to produce the vaccine for LMICs at reasonable quantities and prices.

Two articles proposed alternatives to the PDP model, redirecting PDP funds to build existing public sector pharmaceutical manufacturing capacities in LMICs. Stevenson and Youde [43] and Stevenson [26] noted how this would eliminate the profit motive inherent in PDPs and ensure that demand is met. This approach was leveraged by DNDi, which combined existing HIC public-sector research institution resources and only used pharmaceutical firms as contractors if necessary [26]. In this way, PDPs have duplicated pre-existing public-sector research organisations that share similar mandates yet lack funds. Stevenson [26] noted that by declaring access to preventative and therapeutic health technologies a fundamental tenet of global health policy, governments and enablers of global health partnerships made manufacturers vital to global public health. Efforts could be re-directed to support scaling up existing public-sector health technology production capabilities, for example, in Brazil and Cuba. Meanwhile, PDPs operating outside the direct control of governments and the UN system will continue being relied upon to produce needed health technologies for LMIC populations.

#### GHPs for improved access

Access partnerships include GHPs, such as Gavi, which focus on procuring and delivering vaccine technologies. Four articles discussed Gavi’s accomplishments in increasing access to vaccines in LMICs through AMCs [37, 39, 44, 45]. Jaupart et al. found that Gavi made an important contribution to increasing vaccine uptake in LICs, noting a 12.0% increase in DPT coverage (95% CI 6.6–17.5) and an 8.8% increase in measles-containing vaccine (MCV) coverage (95% CI 3.6–14.0) from its inception until 2016 [45]. This was consistent with findings by Gandhi, who also concluded that Gavi had mixed results in addressing between-country inequities in immunisation uptake [44].

Six articles argued that market-oriented incentives like AMCs were necessary to ensure LMICs obtained adequate quantity, quality, affordability, and sustainability of vaccines [32, 35, 36, 46–48]. Thus, HIC donors channelled development financing into specialised programmes that included AMCs, which they considered could address between-country access inequities [44]. This funding model was initially embraced in 2007 by BMGF, Canada, Italy, Norway, Russia, and the UK, pledging US$ 1.5 billion to accelerate the development of a new pneumococcal vaccine [46].

The premise behind AMCs is that they tackle static and dynamic distortions in the vaccine market through a legally binding commitment to purchase vaccines on predetermined terms. This reduces risks to the pharmaceutical industry and aims to stimulate development and manufacturing of such health technologies [32]. The AMC model sought to address market failure but continues to experience shortcomings in supporting innovative vaccine technologies and price regulation. Though the AMC model successfully incentivises pharmaceutical companies to engage with issues relating to the supply of and demand for vaccines, it has yet to be successful in convincing companies to develop new technologies [38, 49]. This failure is explained by the AMC’s shift from the original intention of developing new vaccine technologies to focusing on increasing production capacity and purchasing and distributing existing vaccines. This shift effectively locked in a price to be paid to vaccine manufacturers, thus providing two multinational producers—GSK and Pfizer, as opposed to vaccine manufacturers located in LMICs such as SII—with a fixed profit level and no incentive to reduce unit prices, with consequences for vaccine accessibility in LMICs [39].

Cernuschi et al. [46] and Siagian and Osorio [49] discussed challenges with the AMC model, such as long-term competition between first- and second-generation vaccine manufacturers. Second-generation suppliers have little incentive to participate as they face technical barriers to market entry [46]. Though supporters of AMCs intended that the model would promote competition to ensure lower prices and avoid supply interruptions, donors did not want to establish differential terms for different suppliers, opting for a “one size fits all” contract [49].

Several articles questioned the sustainability of partnerships, highlighting their heavy donor dependence, especially on Gavi, despite attempting to reassure industry partners about the long-term viability of sustaining vaccine markets in LMICs [38, 48, 49]. Authors suggested exploring less pro-cyclical funding and finding ways for the AMC design to match long-term predictable donor commitments with final buyers’ budgetary capacity [46].

### Critiques of GHPs’ influence on decision-making in global governance

#### BMGF dominance

Several articles identified the creation of BMGF in 2000 as a critical event in the growing role of non-state actors in global health [10, 26, 31, 43]. The enormous resources it commands, dwarfing those from many donor countries and the budgets of some international agencies, give it vast influence. For example, its endowment had already reached US$46.8 billion in 2018, and it had awarded a cumulative US$50 billion in grants and US$3.2 billion in development assistance for health (DAH) in that year. This accounted for over 8% of that year’s global total, making it the third-largest single source of DAH funds after the US and the UK [43]. Unsurprisingly, BMGF is not a passive donor, and its support has focused on specific, often technological, innovations rather than strengthening health systems holistically.

Six articles examined the work of BMGF, highlighting its material, ideational and agenda-setting influences on global health policymaking [10, 26, 32, 42, 43, 50]. Several argued that it weakens international bodies such as WHO and the UN, thus “rendering less relevant multilateral fora where [LMICs] have a voice.” For example, WHO was marginalised in the design and governance of the Access to COVID-19 Tools Accelerator (ACT-A) with traditional multilateral governance—based on national governments—giving way to multi-stakeholder oversight [50]. Yet, while some criticised this development on the grounds of transparency, others argued that it is an understandable response to the “glacial speed” with which many multilateral initiatives progress. In contrast, some GHPs, such as the International AIDS Vaccine Initiative (IAVI), are praised for their more agile response, drawing on techniques more usually associated with the private sector [21, 22, 24].

Stevenson and Youde [43] argued that the influence that BMGF can exert in global health governance is less a reflection of its ability to usurp state power than the abdication of states’ responsibilities. From this perspective, partnerships involving major philanthropic organisations have brought urgently needed resources to bear on complex problems that have long stymied individual governments and the constellation of international actors. This has led governments to look to these arrangements to achieve goals that have proven elusive for the international system. This is exemplified by the support given to GHPs such as Gavi.

Five articles noted how the resources available to GHPs led to their presence at high-level decision-making fora becoming institutionalised [30, 42, 47, 51, 52], despite concerns about their democratic legitimacy and accountability [43]. Commentators contrasted their authority, derived from their resources, with their weak claims to legitimacy. Authority implies a degree of power. Legitimacy refers to the normative right to exercise that authority, requiring the assent of those directly impacted [43]. Some questioned the assumption that private participation in global health is necessary for solving complex problems, given the absence of any mechanism for democratic oversight of internal decision-making in BMGF [43].

#### WHO’s diminishing authority

Several authors considered the impact of GHPs on WHO’s role and influence [10, 25, 26, 43, 50]. As a normative organisation accountable to its member states, WHO’s action is politically constrained [25]. Wenham et al. [25] saw this as an example of a “principal agent relationship”, where WHO’s agency is limited and determined by the compliance and consensus of states, resulting in political interference, whilst also attempting to be an apolitical technical actor. Periodic strengthening initiatives, including the 2005 International Health Regulations revision, have had limited success [10, 50]. In contrast, private philanthropic organisations have much greater freedom, especially those whose authority is concentrated in one or several individuals, such as BMGF. This freedom could make BMGF well-suited to resolve global health challenges. However, the idea that a few private individuals should drive global health policy is problematic, particularly given that WHO’s legitimacy is derived from its 194 member states [43].

GHP funding and interventions have taken a vertical corporatist approach to addressing global health challenges that is incompatible with the more horizontal health systems-oriented approaches endorsed by WHO and countries for achieving vaccine equity. Consequently, evidence suggests that continued usage of GHPs in global health decision-making resulted in the gradual relegation of WHO to peripheral decision-making, acting as a coordinator rather than a leader in governance [26, 43]. Equally, many see approaches based on business models as further excluding LMICs from decision-making processes, exacerbating health technologies access issues [10, 26, 50].

The perceived weakness of UN organisations, such as WHO, has created a gap that non-state actors are attempting to fill. Such moves are often welcomed, given the opportunities for additional resources. As a leading donor and frequent partner, BMGF has demonstrated it will work through WHO and not wait for or defer to WHO to create new initiatives. BMGF justifies this approach by evidencing the success of Gavi and GFATM, showing that alternative organisational structures can provide financial support, promote drug and vaccine development, and build necessary health infrastructure [43]. At the same time, BMGF’s adoption of a public-private partnership model means its goals depend on whether its public sector partners can fulfil their commitments. Stevenson and Youde [43] argued that one of the biggest threats to this model is the unwillingness of BMGF’s government partners to adequately complement its activities, e.g., by scaling up technologies that have been developed or by delivering and implementing these technologies due to insufficient infrastructure in countries. Given its limited capacity and imbalanced resources, some argued that WHO was ill-equipped to enter sustained collaborations with non-state actors, especially the pharmaceutical industry.

Six articles described GHPs’ lack of transparency and accountability [10, 22, 26, 32, 43, 50], noting concerns about the transparency of decision-making processes and the need for global mechanisms to hold GHPs accountable. Lack of transparency and accountability make it difficult to examine the successes and failures of specific interventions [53]. Some also saw this lack as a means to increase private interest representation in decision-making processes. Ruckert and Labonté, in an analysis of 23 GHPs, found that most decision-makers were from the corporate sector and argued that this could create conflicts of interest. For example, BMGF maintains substantial equity in the Coca-Cola Corporation [10], and its grants encourage LMIC communities to become business affiliates of Coca-Cola despite Coca-Cola products contributing to the growing global burdens of obesity and diabetes. Articles have argued that this could partially explain BMGF’s focus on infectious diseases despite NCDs constituting more than half of all deaths in LMICs [10, 43].

#### GHPs create disease-funding silos

GHPs’ focus on product development and delivery for specific diseases undermined horizontal and systems-oriented efforts to reduce disease spread [43]. Three articles described the GHP model as a barrier to addressing the social determinants of health (SDH), such as poverty or social exclusion, resulting in “agenda skewing” [10, 25, 32]. Few GHPs focus on the contribution of SDH to global disease burden. For example, TB shows a strong socioeconomic gradient, with poor and marginalised populations more likely to contract TB due to malnutrition or inadequate access to health services [10]. One article noted how few GHPs have explicit objectives for poverty alleviation or robust means to demonstrate that their interventions benefit poorer populations despite having equity objectives [43]. This is important as certain GHPs working on TB have reduced mortality but been less successful in reducing incidence, reflecting a focus on treatment rather than prevention [43]. Consequently, disease funding silos and segmentation were created, with most international funding directed towards infectious instead of non-infectious diseases. For example, BMGF directs approximately 97% of its financial disbursements towards infectious diseases—with less than 3% to NCDs—and 45% of total PDP funding is from BMGF, resulting in the model’s financial reliance on BMGF [10]. PDPs remain the primary means for incentivising technical innovation and transferring proprietary technologies to actors’ intent on strengthening public health in LMICs [32, 48].

Four articles referred to the increased “financialisation” of the vaccine ecosystem and GHP initiatives’ heightened focus on financial risks to the pharmaceutical industry rather than global public health risks [50, 53–55]. Financialisation is “the rise of financial concepts, motives, practices and institutions” [53]. This approach informed how COVAX approached vaccine production and distribution. Both Stein [53] and Storeng et al. [55] argued that COVAX’s focus on “financialisation” and corporate risk management undermined its goals of achieving global vaccine equity. Both also considered this focus could be the reason behind COVAX’s refusal to support global health policies challenging the global IP regime or corporate privileges, despite providing pharmaceutical companies with both push and pull subsidies. However, this has not benefitted COVAX, as vaccine manufacturers consistently prioritised bilateral deals with wealthier countries instead of supplying COVAX. This was illustrated by vaccine manufacturer Moderna, which reserved most of its doses for bilateral deals with HICs despite receiving funding from CEPI that included conditions on equitable access [55]. However, Moderna only entered an agreement with COVAX in May 2020 for approximately 500 million doses to be delivered in the second half of 2021, and “*only after committing to delivering billions of doses first in bilateral deals*” [55].

## Discussion

### Key findings

This review is unique in its examination of the literature on the role of GHPs in supporting global immunisation mandates, their strengths and limitations, and what these mean for policy, practice, and research. Our analytical themes map the origins and types of GHPs and summarise their key critiques. Despite our broad research question, a limited number of studies were eligible, with even fewer generating empirical data that could shed light on characteristics associated with particular outcomes. This lack of empirical evidence was particularly notable for partnerships seeking to improve access to medicines, such as Gavi, or those involved in pandemic preparedness or response. This lack of data may reflect an inherent lack of accountability of GHPs, with little transparency about decision-making processes–much of the literature comprised case studies, discussion papers, or narrative descriptions.

Eligible studies examined GHPs from different perspectives. Some used an economic lens, seeing GHPs as a response to market failure, with success measured as the ability to stimulate innovation and reduce prices. Studies applying a political lens described a general need for more engagement with LMIC stakeholders in partnerships for access and PDPs. However, PDPs have responded to such criticisms by attempting to work with communities most affected by target diseases. Most authors commended PDPs’ support of capacity-building in regulatory expertise, clinical trial management, and expanding vaccine manufacturing capacity in LMICs. However, two issues arise. First, articles highlighting PDP strengths mainly outlined their successes in supporting regulatory pathways for country-level vaccine approvals where existing vaccine technologies were inaccessible [22, 24, 38, 39]. Of these articles, none identified the PDP model as the main driver in bringing new vaccines to market. DNDi, which brought eight new treatments to the market, achieved some success [39]. However, vaccine production remains complex, and the literature does not indicate whether such vaccines were successfully administered at the population level. Second, despite including LMIC stakeholders in activities, more evidence is needed to determine whether PDPs can propose alternative interventions to speed vaccine innovation, improve access, or create interventions appropriate to local needs [22, 39]. For example, several authors expressed concern about Gavi’s mixed results in addressing between-country inequities in immunisation services because of a lack of necessary national infrastructure [38, 44].

PDPs were seen as duplicating the work of existing public-sector initiatives that shared similar mandates yet had limited funding [26, 32, 34–36]. This underlines an inherent conflict in organisational goals, where public sector partners aim to address population needs within a specific budget, and private sector partners aim to maximise stakeholder profits [32, 34, 35]. Indeed, a public consortium is a logical pathway to circumvent market failure. Still, well-founded doubts about the usefulness of such a strategy have thus far constrained such an arrangement from developing. Countries would have to replicate the billions firms invest in basic research, clinical trials, manufacturing, and distributing end products [26, 32]. Their unwillingness to do so underpins the existing division of labour, whereby basic research is funded publicly. In contrast, the private (though not always for-profit) sector does the rest [26].

Another significant finding relates to using AMCs to spur vaccine innovation and promote competition between first- and second-generation vaccine manufacturers in LMIC markets. The AMC has yet to be able to promote R&D into new vaccines, with its fundamental flaw that it was based on the PCV vaccine, which was already available in the market in some form. Like other GHP approaches to initiating vaccine R&D, the AMC model needs to create opportunities for new technological diffusions for second-generation manufacturers. Articles examining AMC’s successes and failures also noted that it played a role in expanding access to certain vaccines in LMIC markets. Still, gaps remain in global vaccine manufacturing capacity or innovative new vaccine technologies. A lack of transparency on decision-making in GHPs further hampered the AMC model’s success. Though GHPs have an increasing influence in policymaking, they act as brokers between states and vaccine manufacturers, representing both parties’ interests in accelerating access and R&D, mainly in LMICs. There was no evidence to suggest that such models work in HIC markets. Understanding how the GHP model can be leveraged in high-income economies, and GHPs’ role in negotiating prices with vaccine manufacturers on behalf of such economies, will be crucial to understanding whether this model helps achieve global vaccine equity.

This review leaves unanswered questions on the structural factors that influence COVAX’s ability to achieve its goals of global vaccine equity. Though COVAX is a new initiative aiming to increase vaccine availability globally, it has applied existing GHP methods and tools, all of which have only been used in LMIC settings. By contrast, there are numerous other barriers facing COVAX, such as supply shortages and vaccine nationalism. Unlike previous GHP initiatives, COVAX collaborates closely with GHPs, international organisations such as WHO and UNICEF, and public and private sectors to achieve global vaccine equity. COVAX’s mission is to serve both LMIC and HIC populations. However, more research is needed to understand the nature of this partnership, the different levels of power and influence each partner holds, and how they exert such influence to achieve desired outcomes. No studies in this review examined how the individual goals of partners within a GHP may either undermine or support its intended purpose.

### Implications

Critiques of GHPs included in this review suggest they offer numerous advantages to vaccine access and innovation [54]. However, challenges remain, and global vaccine equity has yet to be realised. Both PDPs and access GHPs provide excellent opportunities to address gaps in the vaccine innovation system. Such partnerships are an opportunity to grow and leverage the comparative skills and experiences each partner is uniquely positioned to provide. To make the best of these alliances, we propose the following: (i) increased transparency to provide opportunities for performance and impact evaluation; (ii) refocusing interventions to include both health systems strengthening and cost-effectiveness; and (iii) examination of incentives for cooperative vaccine R&D partnerships and expansion of manufacturing capacity to achieve global immunisation goals.

First, many articles highlighted the need for more transparency among GHPs regarding geographic coverage, funding, governance, and stakeholder involvement. Notably, GHPs have been criticised for the limited engagement of civil society and LMIC stakeholders in decision-making processes, which are dominated by HIC governments and private sector actors. The need for more transparency reduces opportunities to evaluate GHPs and how such characteristics affect their performance and impact. Standardised and routinely collected indicators of pharmaceutical innovation are needed. As such, GHPs should become more transparent, making information about decision-making processes publicly available. As noted by Aerts et al. [48], there is no single, regularly updated, publicly available database that tracks the progress of GHPs on innovation or that catalogues their funding arrangements, geographical scope, R&D spending, and profit margins. Donors could address this by creating a single platform where GHPs would report this information, along with funding received, investments made, and timelines.

Second, all partners in GHPs must be committed to cost-effective policy interventions that strengthen health systems. Private sector involvement in the pursuit of global vaccination goals is essential. However, it has resulted in greater focus on narrow technical interventions rather than systematic strengthening of health systems. GHPs set clear and measurable objectives but often focus on narrow goals, such as the delivery of interventions, rather than the broader goal of strengthening health systems. However, it is important to recognise that this is difficult, particularly establishing attribution, given the many other factors involved.

Finally, the inclusion of equity goals will require GHPs to address the political aspects of the vaccine innovation system. This relates to technology transfer and manufacturing capacity conditions in LMICs, whose governments should be seen as partners rather than aid recipients. Here, the experience with IAVI, developing innovative IP arrangements, offers lessons.

### Limitations

Several limitations should be considered in interpreting our findings. As is common in scoping reviews, one researcher conducted searches and extraction [14]. However, all authors contributed to analysis and interpretation. Interpretation is limited by the absence of a counterfactual against which to compare GHPs and what would have happened if they did not exist. We did not include grey literature (e.g., government or NGO reports) [56] and while these could have provided details of specific GHPs’ organisational planning and policy-related activities it is unclear how much they would add to what was found. Finally, we did not explore some of the other contributions of GHPs, e.g., in areas such as advocacy for TRIPS revisions and greater access to medicines. This could be a topic for future research.

## Conclusion

Our findings contribute to understanding the emergence of GHPs, the mechanisms they have leveraged to support global access to vaccines, and inherent associated challenges. There is little evidence to suggest that GHPs alone can solve the problems of global vaccine equity. By design, GHPs do not explicitly seek to challenge the current economic governance system. Instead, they work within its confines to develop products or increase access by producing technical solutions to political problems. As such, further research is needed to identify opportunities for collaboration among global health governance actors and identify which mechanisms can best ensure successful action towards achieving global vaccine equity.

## Supporting information

S1 Checklist PRISMA checklist. (DOC)

## References

[1] Nguyen A. COVAX Facility governance explained. Gavi, the Vaccine Alliance. 2020.

[2] Mcadams D, Mcdade KK, Ogbuoji O, Johnson M, Dixit S, Yamey G. Incentivising wealthy nations to participate in the COVID-19 Vaccine Global Access Facility (COVAX): a game theory perspective Commentary Vaccine Global Access Facility (COVAX): a game theory perspective. BMJ Global Health. 2020;5:3627. 10.1136/bmjgh-2020-003627 PMC7705419 33257418

[3] Sampat BN, Shadlen KC. The COVID-19 Innovation System. Health affairs (Project Hope). 2021;40(3):400–9. doi: 10.1377/hlthaff.2020.02097 33539184

[4] Wouters OJ, Shadlen KC, Salcher-Konrad M, Pollard AJ, Larson HJ, Teerawattananon Y, et al. Challenges in ensuring global access to COVID-19 vaccines: production, affordability, allocation, and deployment. The Lancet. 2021. doi: 10.1016/S0140-6736(21)00306-8 33587887 PMC7906643

[5] COVAX. COVAX explained 2023 [Available from: https://www.gavi.org/vaccineswork/covax-explained.

[6] Berkley S. COVAX explained. Gavi, the Vaccine Alliance. 2020.

[7] Carlson C. Assessing the impact of global health partnerships. 2004.

[8] Bartsch S. A Critical Appraisal of Global Health Partnerships. In: Rushton S, Williams OD, editors. Partnerships and Foundations in Global Health Governance. London: Palgrave Macmillan UK; 2011. p. 29–52.

[9] Beaglehole R, Bonita R. What is global health? Global health action. 2010;3: doi: 10.3402/gha.v3i0.5142 20386617 PMC2852240

[10] Ruckert A, Labonté R. Public–private partnerships (ppps) in global health: the good, the bad and the ugly. https://doiorg/101080/014365972014970870. 2014;35:1598–614.

[11] Gavi. Gavi launches innovative financing mechanism for access to COVID-19 vaccines. Gavi, the Vaccine Alliance. 2020.

[12] Widdus R. Public-private partnerships: An overview. Transactions of the Royal Society of Tropical Medicine and Hygiene. 2005;99(SUPPL. 1):1. doi: 10.1016/j.trstmh.2005.06.005 16085172

[13] Widdus R. Public-private partnerships for health: their main targets, their diversity, and their future directions. Bulletin of the World Health Organization. 2001;79(8):713–20. 11545327 PMC2566486

[14] Arksey H, O’Malley L. Scoping studies: towards a methodological framework. International Journal of Social Research Methodology. 2005;8:19–32.

[15] Levac D, Colquhoun H, O’Brien KK. Scoping studies: advancing the methodology. Implementation Science. 2010;5:69. doi: 10.1186/1748-5908-5-69 20854677 PMC2954944

[16] Armstrong R, Hall BJ, Doyle J, Waters E. ‘Scoping the scope’ of a cochrane review. Journal of Public Health. 2011;33(1):147–50. 21345890 10.1093/pubmed/fdr015

[17] Wohlin C. Guidelines for Snowballing in Systematic Literature Studies and a Replication in Software Engineering. 2014.

[18] Westphaln KK, Regoeczi W, Masotya M, Vazquez-Westphaln B, Lounsbury K, McDavid L, et al. From Arksey and O’Malley and Beyond: Customizations to enhance a team-based, mixed approach to scoping review methodology. MethodsX. 2021;8:101375. doi: 10.1016/j.mex.2021.101375 34430271 PMC8374523

[19] Reflecting on reflexive thematic analysis, (2019).

[20] Tricco A, Lillie E, Zarin W, O’Brien K, Colquhoun H, Levac D, et al. PRISMA Extension for Scoping Reviews (PRISMA-ScR): Checklist and Explanation. Annals of internal medicine: Ann Intern Med; 2018. p. 467–73. doi: 10.7326/M18-0850 30178033

[21] Hardon A, Blume S. Shifts in global immunisation goals (1984–2004): Unfinished agendas and mixed results. Social Science and Medicine. 2005;60(2):345–56. doi: 10.1016/j.socscimed.2004.05.008 15522490

[22] Chataway J, Smith J. The International AIDS Vaccine Initiative (IAVI): is it getting new science and technology to the world’s neglected majority? World Development (Oxford). 2006;34(1):16–30.

[23] Muraskin W. The global alliance for vaccines and immunization: is it a new model for effective public-private cooperation in international public health? American Journal of Public Health. 2004;94(11):1922–5. doi: 10.2105/ajph.94.11.1922 15514228 PMC1448560

[24] Ahonkhai V, Martins SF, Portet A, Lumpkin M, Hartman D. Speeding Access to Vaccines and Medicines in Low- and Middle-Income Countries: A Case for Change and a Framework for Optimized Product Market Authorization. PloS one. 2016;11(11):e0166515. doi: 10.1371/journal.pone.0166515 27851831 PMC5112794

[25] Wenham C, Busby JW, Youde J, Herten-Crabb A. From Imperialism to the “Golden Age” to the Great Lockdown: The Politics of Global Health Governance. Annual Review of Political Science. 2023;26(1):null.

[26] Stevenson M. PATH: pioneering innovation for global health at the public-private interface. THIRD WORLD QUARTERLY. 2017;38(8):1873–93.

[27] Bank World. World Development Report: Investing in Health. Washington DC: World Bank; 1993. 1993 p.

[28] Chataway J, Brusoni S, Cacciatori E, Hanlin R, Orsenigo L. The International AIDS Vaccine Initiative (IAVI) in a Changing Landscape of Vaccine Development: A Public/Private Partnership as Knowledge Broker and Integrator. European Journal of Development Research. 2007;19(1):100–17.

[29] Huzair F, Borda-Rodriguez A, Upton M. Twenty-first century vaccinomics innovation systems: capacity building in the global south and the role of product development partnerships (PDPs). Special Issue: Vaccines of the 21st Century: vaccinomics for global public health. 2011;15(9):539–43. doi: 10.1089/omi.2011.0036 21732822

[30] Huzair F. The influenza vaccine innovation system and lessons for PDPs. Human Vaccines and Immunotherapeutics. 2012;8(3):398–401. 22327495 10.4161/hv.18701

[31] Moran M, Stevenson M. Illumination and Innovation: What Philanthropic Foundations Bring to Global Health Governance. Global Society. 2013;27(2):117–37.

[32] Mrazek MF, Mossialos E. Stimulating pharmaceutical research and development for neglected diseases. Health Policy. 2003;64(1):75–88. doi: 10.1016/s0168-8510(02)00138-0 12644330

[33] Brooke S, Harner-Jay CM, Lasher H, Jacoby E. How public-private partnerships handle intellectual property: the PATH experience. Intellectual property management in health and agricultural innovation: a handbook of best practices, Volumes 1 and 2. 2007:1755–63.

[34] De Pinho Campos K, Norman CD, Jadad AR. Product development public-private partnerships for public health: a systematic review using qualitative data. Social science & medicine (1982). 2011;73(7):986–94. doi: 10.1016/j.socscimed.2011.06.059 21839562

[35] Jahn R, Muller O, Nost S, Bozorgmehr K. Public-private knowledge transfer and access to medicines: a systematic review and qualitative study of perceptions and roles of scientists involved in HPV vaccine research. Globalization and health. 2020;16(1):22. doi: 10.1186/s12992-020-00552-9 32138789 PMC7059709

[36] Sunyoto T. Partnerships for better neglected disease drug discovery and development: how have we fared? Expert Opinion on Drug Discovery. 2020;15(5):531–7. doi: 10.1080/17460441.2020.1736550 32129688

[37] Mahoney RT. Product Development Partnerships: case studies of a new mechanism for health technology innovation. Health Research Policy and Systems. 2011;9(33). doi: 10.1186/1478-4505-9-33 21871103 PMC3175464

[38] Gilchrist SAN, Nanni A. Lessons learned in shaping vaccine markets in low-income countries: a review of the vaccine market segment supported by the GAVI Alliance. HEALTH POLICY AND PLANNING. 2013;28(8):838–46. doi: 10.1093/heapol/czs123 23174880

[39] Hayter CS, Nisar MA. Spurring Vaccine Development for the Developing World: A Collaborative Governance Perspective on Product Development Partnerships. International Journal of Public Administration. 2018;41(1):46–58.

[40] Turk E, Durrance-Bagale A, Han E, Bell S, Rajan S, Lota MMM, et al. International experiences with co-production and people centredness offer lessons for covid-19 responses. Bmj. 2021;372:m4752. doi: 10.1136/bmj.m4752 33593813 PMC7879267

[41] Bishai DM, Champion C, Steele ME, Thompson L. Product development partnerships hit their stride: Lessons from developing a meningitis vaccine for Africa. Health Affairs. 2011;30(6):1058–64. doi: 10.1377/hlthaff.2011.0295 21653957

[42] Hanlin R, Chataway J, Smith J. Global health public-private partnerships: IAVI, partnerships and capacity building. African journal of medicine and medical sciences. 2007;36 Suppl:69–75. 17703568

[43] Stevenson M, Youde J. Public-private partnering as a modus operandi: Explaining the Gates Foundation’s approach to global health governance. Global Public Health. 2021;16(3):401–14. doi: 10.1080/17441692.2020.1801790 32762617

[44] Gandhi G. Charting the evolution of approaches employed by the Global Alliance for Vaccines and Immunizations (GAVI) to address inequities in access to immunization: a systematic qualitative review of GAVI policies, strategies and resource allocation mechanisms through an equity lens (1999–2014). BMC Public Health. 2015;15(1):1198. doi: 10.1186/s12889-015-2521-8 26621528 PMC4665898

[45] Jaupart P, Dipple L, Dercon S. Has Gavi lived up to its promise? Quasi-experimental evidence on country immunisation rates and child mortality. BMJ Global Health. 2019;4(6):e001789. doi: 10.1136/bmjgh-2019-001789 31908857 PMC6936423

[46] Cernuschi T, Furrer E, Schwalbe N, Jones A, Berndt ER, McAdams S. Advance market commitment for pneumococcal vaccines: putting theory into practice. Bulletin of the World Health Organization. 2011;89:913. doi: 10.2471/BLT.11.087700 22271949 PMC3260895

[47] Mahoney RT, Krattiger A, Clemens JD, Curtiss Iii R. The introduction of new vaccines into developing countries. IV: Global Access Strategies. Vaccine. 2007;25(20):4003–11. doi: 10.1016/j.vaccine.2007.02.047 17363119

[48] Aerts C, Sunyoto T, Tediosi F, Sicuri E. Are public-private partnerships the solution to tackle neglected tropical diseases? A systematic review of the literature. Health Policy. 2017;121:745–54. doi: 10.1016/j.healthpol.2017.05.005 28579276

[49] Siagian RC, Osorio JE. Novel approaches to vaccine development in lower-middle income countries. International Journal of Health Governance. 2018;23(4):288–300.

[50] Legge DG, Kim S. Equitable Access to COVID-19 Vaccines: Cooperation around Research and Production Capacity Is Critical. JOURNAL FOR PEACE AND NUCLEAR DISARMAMENT. 2021;4:73–134.

[51] Buckup S. Global public-private partnerships against neglected diseases: Building governance structures for effective outcomes. Health Economics, Policy and Law. 2008;3(1):31–50. doi: 10.1017/S1744133107004392 18634631

[52] Naimoli JF. Global health partnerships in practice: taking stock of the GAVI Alliance’s new investment in health systems strengthening. INTERNATIONAL JOURNAL OF HEALTH PLANNING AND MANAGEMENT. 2009;24(1):3–25. doi: 10.1002/hpm.969 19165763

[53] Stein F. Risky business: COVAX and the financialization of global vaccine equity. Globalization and Health 2021. doi: 10.1186/s12992-021-00763-8 34544439 PMC8451387

[54] Singh S, Bawa J, Singh B, Singh B, Bika SL. Analyzing GAVI the Vaccine Alliance as a Global Health Partnership Model: A Constructivist Analysis of the Global Health Crisis. Millennial Asia.0(0):09763996221116283.

[55] Storeng KT, de Bengy Puyvallée A, Stein F. COVAX and the rise of the ‘super public private partnership’ for global health. Global Public Health 2021. doi: 10.1080/17441692.2021.1987502 34686103

[56] Schopfel J. Towards a Prague definition of grey literature. 2010.

